# Analysis of pregnancy outcomes following surgical treatment of cesarean scar pregnancy

**DOI:** 10.1186/s12884-022-04965-9

**Published:** 2022-08-16

**Authors:** Zongxu Xu, Chengcheng Sheng, Qing Yang, Jun Wang

**Affiliations:** Department of Obstetrics and Gynecology, Shengjing Hospital of China Medical University, No. 36, Sanhao Street, Heping District, Shenyang City, Liaoning Province, 110004 People’s Republic of China

**Keywords:** Cesarean scar pregnancy, Subsequent pregnancy, Follow-up

## Abstract

**Purpose:**

To investigate the surgical treatment approaches for patients with Cesarean scar pregnancy (CSP) and the effects on subsequent pregnancy.

**Methods:**

CSP patients admitted to Shengjing Hospital of China Medical University from January 2013 to December 2018 were retrospectively analyzed to collect their clinical characteristics, and follow-up of postoperative pregnancies.

**Results:**

A total of 1126 CSP patients were enrolled in this study, including 595 (52.84%) CSP type I, 415 (36.86%) CSP type II, and 116 (10.30%) CSP type III cases. There were significant differences between the three types of patients in terms of β-HCG levels, gestational sac diameter, clinical symptoms and presence of fetal heartbeat at diagnosis (*P* < 0.01). Among these, 89.90% of CSP type I, 88.90% of CSP type II and 50% of CSP type III patients were treated with hysteroscopic lesion excision, 7.9% of CSP type I and 2.2% of CSP type II patients underwent ultrasound-monitored curettage, and the remaining patients underwent lesion excision and and simultaneous repair of excised lesions by different routes (trans-laparoscopic, transabdominal or transvaginal methods). And 5.55% of CSP type I, 22.65% of CSP type II and 43.10% of CSP type III patients were treated with adjunctive uterine artery embolization (UAE). The patients were followed up for more than 2 years after surgery. Among the 166 re-pregnancies, 58 (34.94%) were normal pregnancies, 17 patients reoccurred with CSP, the recurrent rate of CSP was 10.24%. All 58 normal pregnancies were terminated by cesarean section, with a mean gestational week of delivery of (38.36 ± 2.25) weeks, a mean birth weight of (3228.45 ± 301.96)g, and the postnatal Apgar score was (9.86 ± 0.23) points at 1 min and all 5 min were 10 points. Logistic regression analysis suggested that the number of previous cesarean deliveries was a risk factor for recurrent CSP (RCSP) (OR = 10.82, 95% CI: 2.52–46.50, *P* = 0.001).

**Conclusions:**

The type of CSP is related to β-HCG values, presence of fetal heartbeat, gestational sac diameter and clinical symptoms. Hysteroscopic therapy is a commonly used surgical procedure and UAE is often used as an adjuvant treatment. For subsequent pregnancies, the number of previous cesarean deliveries is a risk factor for recurrent CSP.

## Background

Cesarean scar pregnancy (CSP) is a special type of ectopic pregnancy, which refers to the implantation of gestational sac lays within the scar of a previous cesarean delivery and is one of long-term complications of cesarean delivery [1]. The incidence of CSP is increasing due to the increasing in the rate of cesarean delivery and advances in imaging techniques in recent years [2, 3]. Studies have reported that the incident of CSP is about 1:1800 to 1:2500 in all pregnancy, while among women with a history of at least one cesarean delivery, CSP accounts for 6.1% of all ectopic pregnancies [4, 5]. The early symptoms of CSP are atypical, and when CSP is not diagnosed in time, threatened abortion or curettage occurs, resulting in incomplete abortion, which often leads to vast vaginal bleeding and even endangers the patient's life [6, 7]. When CSP continues without timely diagnosis and treatment, there is a risk of placental implantation, postpartum hemorrhage or even uterine rupture, which requires hysterectomy and loss of reproductive function if necessary [8, 9], therefore, it is recommended that CSP should be terminated immediately upon diagnosis [10].

Different treatment options have been developed in the course of continuous research on CSP, and it has been explored by scholars in various countries to choose the appropriate treatment option that leads the least damage and costs and does not affect the long-term quality of life of the patient. In this study, we analyzed the general profile of CSP patients admitted to our hospital from 2013 to 2018, and these patients were followed up for more than two years and their subsequent pregnancy outcomes were analyzed. By comparing treatment options for different CSP types and the impact on following reproduction, we hope to provide suggestions for the future treatment of CSP patients.

## Methods

### Case collections

We retrospective collected patients hospitalized for surgical treatment with the diagnosis of CSP in Shengjing Hospital of China Medical University, a tertiary referral center in Liaoning China from January 2013 to December 2018. CSP was diagnosed on the basis of pelvic ultrasound or magnetic resonance imaging (MRI) images archived in the electronic medical record system, according to the criteria proposed by Vial et al., which included a void in the uterine cavity and cervical canal, and a gestational sac lodged in the lower myometrium of the anterior uterine wall (equivalent to the site of a previous cesarean section), and a visible defective thinning of the myometrium between the gestational sac and the bladder [11]. All cases were first-visit cases with complete medical records and were followed up by telephone for two years or longer, and follow-up completion is in early 2021. A total of 1684 patients were included in this study, and 473 were lost due to communication interruptions and address changes, and 85 were excluded because they did not meet the inclusion criteria, so 1126 patients were included in the analysis. CSP was classified into three types according to the "Expert consensus on the diagnosis and management of Cesarean scar pregnancy" of the Family Planning Group of the Obstetrics and Gynecology Branch of the Chinese Medical Association (Table 1) [12].

**Table 1:** CSP types and diagnostic criteria [12]

Types	Criteria
I	Partial implantation of the gestational sac in the uterine scar, thinning of the myometrium between the gestational sac and the bladder, thickness > 3 mm
II	Partial implantation of the gestational sac in the uterine scar, thinning of the myometrium between the gestational sac and the bladder, thickness ≤ 3 mm
III	Completely implantation of the gestational sac in the uterine scar and convex toward the bladder; or a mixed echogenic mass be seen in the lower uterine scar, with the mass bulging toward the bladder, CDFI shows a rich blood flow signal around the mass; significantly thinning or even absent of the myometrium between the gestational sac and the bladder, thickness ≤ 3 mm

Inclusion criteria: (1) history of at least one previous cesarean delivery; (2) women of age less than 45 years; (3) postoperative pathology suggested a normal pregnancy with villi; (4) consistent with the diagnostic criteria of CSP: fertilized eggs lodged in the uterine incision scar of the previous cesarean delivery in early pregnancy (≤ 12 weeks).

Exclusion criteria: (1) gestational week > 12 weeks; (2) uterine scar pregnancy combined with a heterotropic pregnancy; (3) simultaneous hysterectomy or sterilization and loss of natural ability to conceive; (4) Severe postoperative uterine adhesions that may affect conception; (5) referral cases after poor treatment in other hospitals.

### Data collections

The electronic medical record system of Shengjing Hospital of China Medical University was checked to collect information on the patients’ age, previous pregnancy history, months since last cesarean delivery (m), gestation age (d), symptoms, Serum beta human chorionic gonadotropin (β-HCG) values before treatment, presence of fetal heartbeat, maximum diameter of the gestational sac and treatment methods.

### Treatment methods

- (1) Surgical treatment: ultrasound-monitored curettage, hysteroscopic lesion excision, and excision and repair of lesions through different routes (including transabdominal, trans-laparoscopic and transvaginal methods).
- (2) Adjunctive treatment: bilateral uterine artery embolization (UAE), which involves the embolization of bilateral uterine arteries with gelatin sponge particles injected under intervention. UAE can be used as an emergency treatment in case of hemorrhage or as a pretreatment to reduce intraoperative or postoperative bleeding in CSP [13].

### Follow-up

All enrolled patients were followed up by telephone for a minimum of two years with information on subsequent pregnancy, including interval months between second pregnancy and CSP treatment, pregnancy outcome, mode of delivery and neonatal outcome.

## Statistical analysis

SPSS 24.0 was used for statistical analysis, descriptive statistics are shown as the standard deviation of the mean, percentage, and frequency, using t-test, Mann–Whitney U test, or a chi-square test. Logistic regression analysis was performed for potential factors affecting the recurrent CSP. *P* < 0.05 indicates statistically significant.

## Results

### General clinical characteristics of the enrolled patients

**Fig. 1:**
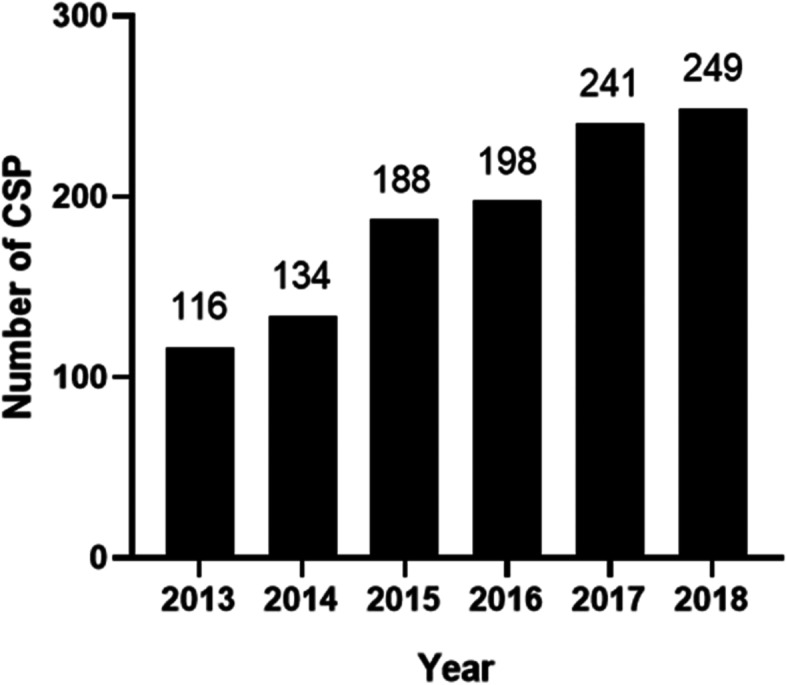
Number of enrolled cesarean scar pregnancies at Shengjing Hospital of China Medical University during the years from 2013 to 2018. CSP, Cesarean scar pregnancy

The total number of patients eligible for inclusion in this study was 1126. As showing in Fig. 1. the number of patients with CSP enrolled in this study increased year by year. The general clinical characteristics of all the patients enrolled is shown in Table 2. with average age at diagnosis (32.97 ± 4.49) years. The time since previous cesarean delivery was (76.42 ± 49.59) months, and the gestational age was (67.33 ± 3.30) days. The 39.79% of patients had a vaginal bleeding, while 8.26% cases had abdominal pain. 186 (16.52%) cases had both abdominal pain and bleeding and 399 (35.43%) cases were asymptomatic. 1126 patients were divided into three groups according to the criteria in Table 1, and 52.84% were CSP type I, 36.86% were CSP type II and 10.30% were CSP type III.

**Table 2:** Clinical characteristics of 1126 patients with CSP

Characteristic	Values
Age (years)	32.97 ± 4.49
Number of prior pregnancies	3.77 ± 1.46
Number of abortions	1.27 ± 1.15
Number of prior cesarean delivery	1.60 ± 0.49
Time since previous cesarean delivery(Months)	76.42 ± 49.59
Symptoms at diagnosis	
Abdominal pain	93(8.26)
Vaginal bleeding	448(39.79)
Both	186(16.52)
None	399(35.43)
Gestational age(days)	67.33 ± 3.30
β-HCG level at diagnosis (mIU/mL)	20,494.00 ± 8701.00
Gestational sac size(cm)	2.93 ± 0.83
Presence of fetal heartbeat	
Yes	395(35.08)
No	731(64.92)
Types of CSP	
I	595(52.84)
II	415(36.86)
III	116(10.30)

Data are n, mean ± SD, or n (%)

*CSP* cesarean scar pregnancy, *β-HCG* Beta-human chorionic gonadotropin

### Comparison of clinical characteristics among three groups with different types of CSP

The CSP patients was divided into three groups according to the CSP types in Table 3. There was no statistical difference among the three groups in terms of age at diagnosis, number of pregnancies, number of abortions, number of cesarean deliveries, time since last cesarean delivery and the gestational age. The symptoms were statistically different between the three groups, and with a weak correlation between them(Cramer’s V = 0.097, *P* = 0.002). There were significant differences among the three groups in terms of serum β-HCG levels at diagnosis and maximum diameter of the gestational sac(*P* < 0.05). The mean serum β-HCG levels were significantly higher in CSP type II and CSP type III than that in CSP type I, and CSP type III had the largest gestational sac diameter (3.50 ± 1.49) cm. The proportion of patients with fetal heart beats was significantly higher in CSP type I than in CSP type II and CSP type III(*P* < 0.05).

**Table 3:** Comparison of clinical characteristics among three groups with different types of CSP

**Characteristic**		**CSP Types**		***P***
	**I(*n* = 595)**	**II(*n* = 415)**	**III(*n* = 116)**	
Age (years)	33.02 ± 4.39	33.12 ± 4.67	32.07 ± 4.25	0.170
Number of prior pregnancies	3.76 ± 1.44	3.83 ± 1.45	1.14 ± 0.34	0.090
Number of abortions	1.23 ± 1.10	1.31 ± 1.21	1.04 ± 1.28	0.480
Number of prior cesarean delivery	1.21 ± 0.44	1.22 ± 0.42	1.14 ± 0.34	0.190
Time since previous cesarean delivery(Months)	79.36 ± 50.03	72.90 ± 48.53	73.90 ± 50.15	0.330
Symptoms at diagnosis				
Abdominal pain	60(10.08)	26(6.26)	7(6.03)	
Vaginal bleeding	216(36.30)	180(43.37)	52(44.83)	
Both	116(19.50)	48(11.57)	22(18.97)	
None	203(34.12)	161(38.80)	35(30.17)	0.002*
Gestational age(days)	53.30 ± 10.82	54.03 ± 10.42	55.65 ± 12.14	0.100
β-HCG level at diagnosis (mIU/mL)	20,494.00 ± 8701.00	63,494.18 ± 72,904.21^ad^	59,749.33 ± 56,315.99^ cd^	0.000*
Gestational sac size(cm)	2.35 ± 0.15	2.94 ± 1.36^ad^	3.50 ± 1.49^bcd^	0.000*
Presence of fetal heartbeat				
Yes	403(67.73)	166(40.00)	37(31.90)	
No	192(32.27)	249(60.00)^ad^	79(68.10)^cd^	0.000*

Data are n, mean ± SD, or n (%)

CSP, cesarean scar pregnancy; β-HCG, Beta-human chorionic gonadotropin

“*” indicates a statistically significant difference

^a^group I vs. group II

^b^group II vs. group III

^c^group I vs. group III

^d^*P* < .05

### Comparison of treatment modalities for patients with different types of CSP

An analysis of the different surgical treatments for CSP revealed that hysteroscopic lesion excision was the most common surgical treatment, with 89.90% of CSP type I patients, 88.90% of CSP type II patients and 50.00% of CSP type III patients undergoing this surgical procedure. Ultrasound-monitored curettage was utilized in 7.9% of CSP type I patients compared with 2.2% in the CSP type II patient group, and no one in CSP type III group. 50% of CSP type III patients underwent lesion excision and repair operation, including 14.66% through trans-laparoscopic, 13.79% through transabdominal approach, and 21.55% through transvaginal approach. There was a significant difference in the surgical treatment approach taken by the three groups of CSP patients (*P* < 0.01) (Table 4).

**Table 4:** Comparison of surgical treatment among three groups with different types of CSP

Surgical treatment	Types of CSP		
	**I(*n* = 595)**	**II(*n* = 415)**	**III(*n* = 116)**
Hysteroscopic lesion excision	535(89.90)	369 (88.90)	58 (50.00)
Ultrasound-monitored curettage	47(7.90)	9 (2.20)	0 (0)
Laparoscopic lesion excision	2(0.30)	9 (2.20)	17 (14.66)
Transabdominal lesion excision	0(0.00)	7 (1.70)	16 (13.79)
Transvaginal esion excision	11(1.8)	21 (5.10)	25 (21.55)
***P***	0.000*		

Data are n (%)

CSP, cesarean scar pregnancy

“*” indicates a statistically significant difference

Uterine artery embolization (UAE) is often used as an adjunctive treatment for obstetric and gynecologic massive hemorrhage disorders. Table 5 shows the combination application of UAE in the treatment of three groups of CSP patients, with 43.1% of CSP type III patients receiving UAE in combination, much higher than 22.65% in the CSP type II group and 5.55% in the CSP type I group, there was a significant difference among three groups (*P* < 0.01). As to the analysis of various operation methods, there was significant difference in the three types of CSP in hysteroscopic lesion excision group and transvaginal lesion excision group (*P* < 0.01).

**Table 5:** Comparison of combined UAE treatment in patients with CSP of different surgical treatment

UAE treatment	Types of CSP			*P*
	**I(*n* = 595)**	**II(*n* = 415)**	**III(*n* = 116)**	
N				
Yes	33(5.55)	94 (22.65) ^ad^	50(43.10)^bcd^	0.000*
No	562(94.45)	321 (77.35)	66(56.90)	
Hysteroscopic lesion excision				
Yes	26(4.86)	84 (22.76) ^ad^	35(60.34)^bcd^	0.000*
No	509(95.14)	285 (77.24)	23(29.66)	
Ultrasound-monitored curettage				
Yes	7(14.89)	5 (55.56)	0	-
No	40(85.11)	4 (44.44)	0	
Laparoscopic lesion excision				
Yes	0	2 (22.22)	2(11.76)	0.050
No	2(100.00)	7 (77.78)	15(88.24)	
Transabdominal lesion excision				
Yes	0	2 (28.57)	4(25.00)	-
No	0	5 (71.43)	12(75.00)	
Transvaginal esion excision				
Yes	0	1 (4.76)	9(36.00)	0.002*
No	11(100.00)	20 (95.24)	16(64.00)	

Data are n (%)

CSP, cesarean scar pregnancy; UAE, uterine artery embolization

“*” indicates a statistically significant difference

^a^group I vs. group II

^b^group II vs. group III

^c^group I vs. group III

^d^*P* < .05

### Follow-up of subsequent pregnancy in CSP patients

#### Comparison of patients with normal pregnancy and patients with recurrent CSP

**Fig. 2:**
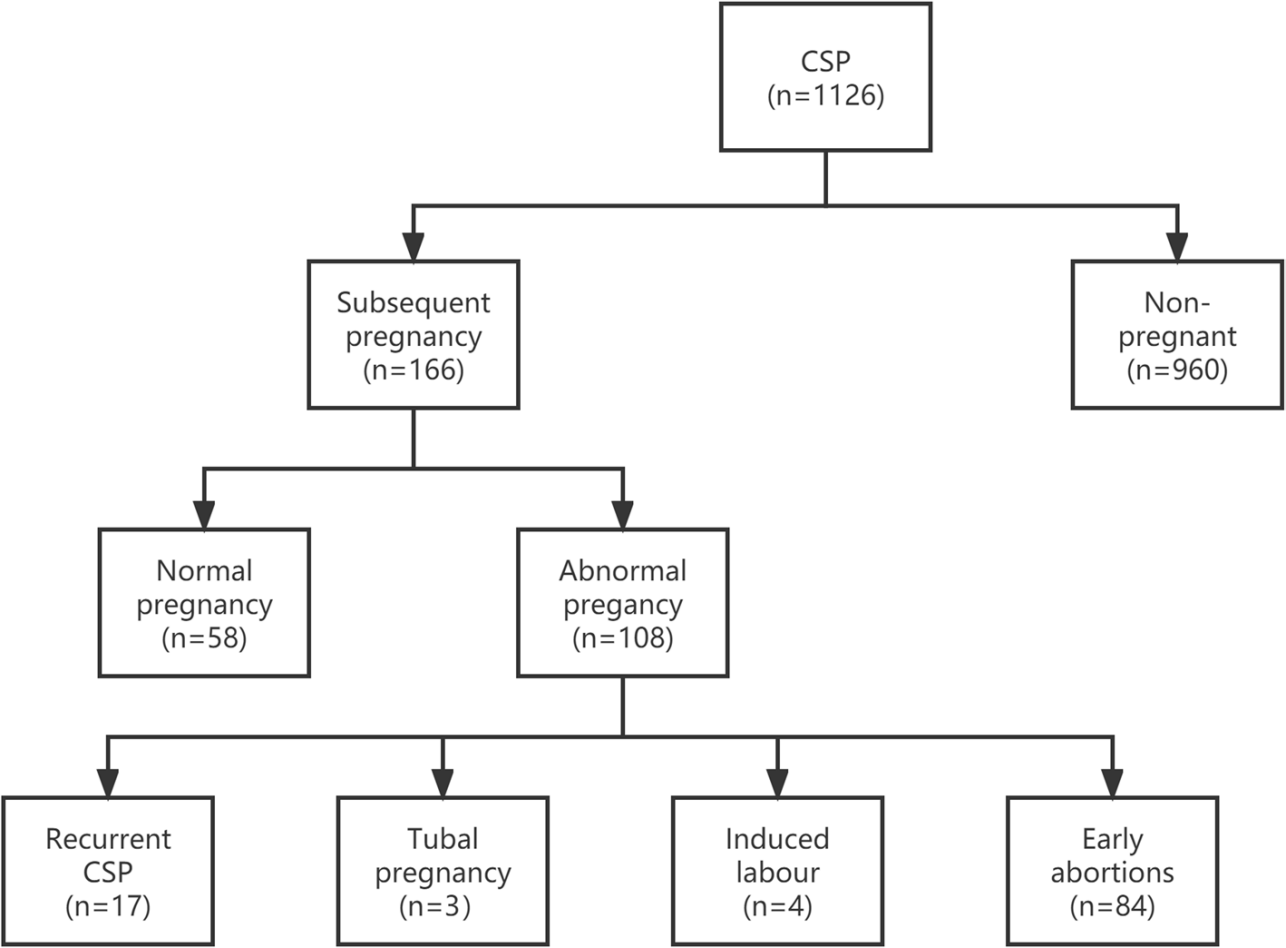
Follow-up of subsequent pregnancy after CSP treatment in patients enrolled in this study. CSP, Cesarean scar pregnancy

All the enrolled patients were followed up for at least 2 years after surgery for the subsequent pregnancy. As shown in Fig. 2, 960 (85.26%) patients did not have another pregnancy during the follow-up period. 166 patients had a subsequent pregnancy, including 58 (34.94%) patients with normal pregnancy. Abnormal pregnancy was seen in 108 (65.06%) patients, of which 17 patients had recurrent CSP (RCSP), the recurrence rate of CSP was 10.24%, 3 (1.82%) patients had tubal ectopic pregnancy, 84 (50.60%) patients had artificial abortion in early pregnancy due to unplanned pregnancy or spontaneous abortion, and 4 (2.40%) cases had induction of labour in mid/late pregnancy due to fetal abnormality or intrauterine death.

To analyze risk factors for recurrent CSP, we compared the clinical characteristics of previous CSP in the normal pregnancy group and the recurrent CSP group. As shown in Table 6, the number of cesarean deliveries and the symptoms at diagnosis of the first CSP were significantly different in the recurrent CSP group from those in the normal pregnancy group (*P* < 0.01). By logistic regression analysis, the number of previous cesarean deliveries was significantly associated with the recurrence of CSP and was a risk factor for RCSP (OR = 10.82, 95% CI: 2.52–46.50, *P* = 0.001) (Table 7).

**Table 6:** Comparison of clinical characteristics of patients with normal pregnancy and patients with recurrent CSP after CSP surgery

Characteristics	Normal pregnancy(*n* = 58)	Recurrent CSP(*n* = 17)	*P*
Age (years)	32.31 ± 4.19	32.47 ± 3.50	0.890
Number of prior pregnancies	3.50 ± 1.61	3.88 ± 1.17	0.116
Number of abortions	1.19 ± 1.29	1.24 ± 1.25	0.811
Number of prior cesarean delivery	1.07 ± 0.26	1.41 ± 0.49	0.000*
β-HCG level at diagnosis (mIU/mL)	48,781.43 ± 45,648.66	71,806.06 ± 65,291.07	0.333
Gestational sac size(cm)	2.72 ± 1.31	2.67 ± 1.00	0.834
Gestational age(days)	52.62 ± 8.82	56.18 ± 11.07	0.257
Presence of fetal heartbeat			0.845
Yes	19(32.76)	6(35.29)	
No	39(67.24)	11(64.71)	
Symptoms at diagnosis			0.001*
Abdominal pain	2(3.45)	0(0.00)	
Vaginal bleeding	26(44.83)	7(41.18)	
Both	6(1.34)	4(23.53)	
None	24(41.38)	6(35.29)	
Types of CSP			0.793
I	31(53.45)	8(47.06)	
II	18(31.03)	7(41.18)	
III	9(15.52)	2(11.76)	
**Surgical treatment**			0.292
Hysteroscopic lesion excision	52(89.66)	13(76.47)	
Ultrasound-monitored curettage	4(6.90)	2(11.76)	
Laparoscopic lesion excision	1(1.72)	1(5.88)	
Transabdominal lesion excision	0	1(5.88)	
Transvaginal esion excision	1(1.72)	0	
UAE treatment			0.172
Yes	9(15.52)	5(29.41)	
No	49(84.48)	12(70.59)	
Time since CSP treatment(Months)	19.07 ± 12.72	25.18 ± 20.05	0.322

Data are n, mean ± SD, or n (%)

CSP, cesarean scar pregnancy; β-HCG, Beta-human chorionic gonadotropin; UAE, uterine artery embolization

“*” indicates a statistically significant difference

**Table 7:** Logistic regression analysis of risk factors for recurrent CSP

Variables	OR	95%CI	*P*
Number of prior cesarean delivery	10.82	2.52–46.50	0.001*
Symptoms at diagnosis			
Abdominal pain	1.00		
Vaginal bleeding	-		
Both	1.16	0.30–4.54	0.832
None	3.92	0.71–21.56	0.116

CSP, cesarean scar pregnancy

“*” indicates a statistically significant difference

#### Pregnancy status of normal pregnant patients after CSP

Subsequent pregnancy outcomes of patients with normal pregnancy were shown in Table 8. Among the 58 normal pregnancies after CSP, 57 (98.28%) were conceived naturally and 1 (1.72%) was conceived by in vitro fertilization and embryo transfer due to tubal occlusion. Four of the 58 patients developed gestational diabetes mellitus, two developed hypertensive disorders, two combined placenta praevia but the placenta was located in the posterior wall, and one developed premature rupture of membranes in late pregnancy. The mean gestational weeks were (38.36 ± 2.25) weeks, the mean birth weight was (3228.45 ± 301.96) g, and the postnatal Apgar score was (9.86 ± 0.23) points at 1 min and all 5 min were 10 points.

**Table 8:** Outcomes of subsequent pregnancy of patients with normal pregnancy after CSP

Characteristics	Values
Conception methods	
Natural conception	57(98.28)
Assisted conception	1(1.72)
Gestational weeks	38.36 ± 2.25
Delivery methods	
Cesarean delivery	58(100.00)
Transvaginal delivery	0
Newborn birth weight(g)	3228.45 ± 301.96
Apgar score	
1 min	9.86 ± 0.23
5 min	10
Complications during pregnancy	
Gestational hypertension	2(3.45)
Gestational diabetes mellitus	4(6.90)
Placenta praevia	2(3.14)
Premature rupture of membranes	1(1.72)

Data are n, mean ± SD, or n (%)

CSP, cesarean scar pregnancy

## Discussions

CSP is a special type of ectopic pregnancy and is a serious long-term complication of cesarean delivery. The incidence of CSP is relatively low, but due to the high rate of cesarean delivery in China in recent years, the occurrence of CSP is also on the rise, and the yearly increase of enrolled patients in this study reflects this situation. CSP occurs mostly in women of childbearing age between 20 and 45 years old, and if not diagnosed and treated in time, it may lead to hemorrhage or even rupture of the uterus. Ideal management of CSP should be prompt diagnosis, active and effective treatment to avoid life-threatening hemorrhage and protect reproductive function [14].

The risk and treatment difficulty of different CSP patients is different. According to the invasion degree of the gestational sac within the scar, CSP was classified into types I to III by the Chinese Medical Association Family Planning Group [12]. Referring to this criterion, we classified the patients in our research and found that the patients were predominantly CSP type I, accounting for 52.84%, while the most dangerous CSP type III was also present in about 1/10, and therefore should not be ignored. There was a significant difference in the clinical symptoms among the three groups, with more patients with CSP type I presenting with abdominal pain and more patients with CSP type II and type III presenting with bleeding.

The results of different studies regarding the relationship between serum β-HCG levels, fetal sac size and CSP types and treatments are inconsistent. Takashi Mitsu et al. found that patients with CSP with low blood β-HCG levels at diagnosis and small gestational sac diameter had a higher success rate with conservative treatment [15]. However, Ni-Chin Tsai et al. concluded that in patients with CSP, blood β-HCG levels and gestational sac diameter did not correlate significantly with the degree of placental implantation. High levels of blood β-HCG do not imply a reduced success rate of conservative treatment [16]. In the present study, patients with CSP type II and III had higher blood β-HCG levels and sac diameter than patients with CSP type I, which may be due to the fact that blood β-HCG levels and gestational sac size suggest different periods of pregnancy, reflecting a more abundant blood flow around the gestational sac, and correlate to some extent with the severity of CSP [17]. However, both types II and III of CSP group in our study were combined with significantly reduced ratio of fetal heart beats, it might be due to that scarred site of gestational sac implantation was unsuitable for embryonic development.

The choice of CSP treatment should consider CSP types, fertility requirements, operator habits and even the cost of the surgery, and requiring a comprehensive consideration for a personalized regime [4]. Ultrasound-monitored curettage is the least expensive option for CSP treatment, but can be tricky if massive bleeding occurring during the operation. In our study, only 7.90% of patients with CSP type I and 2.2% of patients with CSP type II underwent ultrasound-monitored curettage, and nearly 90% of the CSP type I and II patients and half of the CSP type III patients underwent hysteroscopic lesion excision. In a review by Kanat-Pektas, which included 1647 patients with CSP, it was noted that Cesarean scar pregnancy lesion excision (including transabdominal/trans-laparoscopic/transvaginal surgery) had the highest success rate and the lowest hysterectomy rate, followed by Hysteroscopic lesion resection [18]. In this study, 2.2% of CSP type I patients, 8.92% of type II and 50% of type III patients underwent different routes of focal lesion excision and repair. Hasegawa suggested repairing the uterine scar to reduce the risk of recurrence of CSP [19], but Ben Nagi argued against it, claiming that surgical repair of the defect at the scar may bring more complications and may do more harm for women who wish to preserve their reproductive function [20]. The available evidence supports surgical treatment as the treatment of choice over pharmacological treatment for CSP, and although the best treatment option for CSP cannot be determined yet, all five treatment options in this paper are recommended [21].

UAE is a minimally invasive vascular intervention technique to block blood flow through the femoral artery by injecting an embolic agent into the uterine artery. This technique is less invasive, reliable in hemostasis, and is increasingly used in the non-surgical treatment of obstetric and gynecological hemorrhage and uterine fibroids, but the short-term and long-term complications of UAE still deserve attention. Cao et al. showed that UAE may lead to impaired endometrial and ovarian function, resulting in reduced menstrual flow or even amenorrhea [22]. Another study suggested that the use of UAE may lead to a decrease in the rate of natural pregnancy and an increased risk of spontaneous abortion [23]. Therefore, we believe that UAE should be used cautiously in the treatment of CSP, as preoperative pretreatment when the risk of major bleeding is high, or as an emergency hemostatic measure for CSP. In the present study, UAE was mainly used for adjuvant therapy of CSP type II and type III, which was used in only 21 cases as emergency management of massive vaginal bleeding, 19 of which were used on emergency admission and 2 were used for massive vaginal bleeding after hysteroscopy. The rest were used preoperatively prophylactically to prevent heavy intraoperative and postoperative bleeding.

To observe the effect of different treatments on the subsequent pregnancy of CSP patients after surgery, we performed a follow-up of more than two years and only 166 women had another pregnancy, and 960 (85.26%) patients did not during the follow-up period. Most patients had no intention to prepare for another pregnancy because they already had one or more children, and some even opted for contraception for fear of another scarred pregnancy. In this study, 84 patients who had failed in contraception opted for an early abortion. Among the patients who had another pregnancy, 17 cases had a reoccurrence of CSP, and the incidence of RCSP was 10.24%, which is close to that in the Tang study [24]. Qian's study suggested that lower grade of hospital for the previous cesarean section, thinness of the lower uterine musculature before pregnancy and the presence of combined abdominal pain and vaginal bleeding at the previous CSP were risk factors for the occurrence of RCSP [25]. In this study, we also compared the previous CSP clinical characteristics between the normal pregnancy group and the RCSP group and found that there were no significant differences between the two groups in terms of previous CSP types, surgical treatments, and whether UAE was used in combination, but the number of previous cesarean deliveries was significantly higher in the RCSP group than in the normal pregnancy group, and the proportion of symptoms such as both of abdominal pain and bleeding occurred in previous CSP was significantly higher in the RCSP group. Logistic regression analysis suggested that the number of previous cesarean deliveries was a risk factor for RCSP, which significantly elevated the risk of RCSP (OR = 10.82, 95% CI: 2.52–46.50, *P* = 0.001).

There is no uniformity in the interval between re-pregnancies after CSP, and domestic experts recommend an interval of at least six months before another pregnancy [12]. The shortest interval between normal pregnancy and the CSP surgery in this study was 4 months, the longest was 48 months, and the average was 19 months. Normal pregnancy after CSP treatment was not significantly related to either CSP typing or surgery treatment, in agreement with the findings of Sun, Xu et al. [26, 27].

This study analyzed CSP cases occurring in a regional medical center in Northeastern China from 2013–2018, and found that the number of CSP patients admitted to the hospital increased yearly. The CSP cases in this center were predominantly type I, and the main treatment option is hysteroscopic surgery to remove the lesion. As a retrospective research there are some limitations in the present study. The shortest observed cases in this study were only followed up to 2 years, and more than 85% of the patients had not conceived again, and there were not many cases followed up for the outcome of another pregnancy. The subjects were all from comprehensive tertiary care hospitals, with a degree of selective bias. Future large prospective studies recruiting more patients with longer follow-up from multiple centers are necessary to validate our findings.

## Conclusions

In summary, the incidence of CSP has shown a significant upward trend in recent years. There are significant differences in β-HCG values, presence of fetal heartbeat, gestational sac diameter and clinical symptoms between the different types of CSP. The surgical approach is different and treatment needs to be tailored to the CSP types and the specific situation. Hysteroscopic treatment plays an important role in the surgical management of cesarean scar pregnancy, and UAE can be used as an adjuvant treatment for patients at high risk of bleeding. For subsequent pregnancies, the number of previous cesarean deliveries is a risk factor for recurrent CSP. All 58 normal pregnancies had a satisfactory pregnancy outcome without uterine rupture.

## Data Availability

The datasets used and/or analysed during the current study are available from the corresponding author on reasonable request.

## Abbreviations

CSP: Caesarean scar pregnancy
MRI: Magnetic resonance imaging
MTX: Methotrexate
UAE: Uterine artery embolization
β-HCG: Beta-human chorionic gonadotropin
